# The first record of the thomisid genus *Ibana* Benjamin, 2014 (Araneae, Thomisidae) from China, with the description of a new species

**DOI:** 10.3897/BDJ.10.e93637

**Published:** 2022-09-29

**Authors:** Keke Liu, Wenhui Li, Yanbin Yao, Congzheng Li, Shuqiang Li

**Affiliations:** 1 College of Life Science, Jinggangshan University, Ji'an, China College of Life Science, Jinggangshan University Ji'an China; 2 Jinshan College of Fujian Agriculture And Forestry University, Fuzhou, China Jinshan College of Fujian Agriculture And Forestry University Fuzhou China; 3 Institute of Zoology, Chinese Academy of sciences, Beijing, China Institute of Zoology, Chinese Academy of sciences Beijing China

**Keywords:** taxonomy, spiders, types, Jiangxi

## Abstract

**Background:**

The genus *Ibana* Benjamin, 2014 was established, based on the type species *Ibanasenagang* Benjamin, 2014 from Borneo, Malaysia. No species of this genus has been recorded from China.

**New information:**

A new species of *Ibana* is described and illustrated, representing the first record of the genus for China. *Ibanagan* **sp. n.** differs from its congener by the yellowishbrown longitudinal band on the abdomen and the round, contiguous spermathecae. The distribution of the new species in Jiangxi Province is mapped.

## Introduction

The spider family Thomisidae Sundevall, 1833 includes 171 genera and 2167 species worldwide (WSC 2022). In China, 311 thomisid species belonging to 52 genera are known (WSC 2022). Although China has the most species- and genus-rich thomisid fauna (Liu et al. 2017, Li 2020, Liu et al. 2022, Hong et al. 2022, Zhu et al. 2022), not much attention has been paid to their systematics in this country. During the past ten years, only a few new species have been discovered from China. Liu et al. (2022) identified 34 thomisid species, including five new ones, in the Jinggang Mountain National Nature Reserve in Jiangxi Province (Liu et al. 2022), which suggests a high diversity of this group in this region.

*Ibana* Benjamin, 2014 is currently monotypic with its type species, *Ibanasenagang* Benjamin, 2014, recorded from Borneo, Malaysia. However, the male and female of this species were collected on different time and localities (Benjamin 2014) and grouped, based on appearance. Benjamin (2014) characterised the genus by the male palp with a small conductor, the sperm duct with an inward turn, hook-shaped tibial apophysis and the presence of more than three thick long spines on the tibia and the epigyne with kidney-shaped spermathecae and long copulatory ducts (Benjamin 2014).

Jinggang Mountain is located in the middle section of Luoxiao Mountains and on the common boundary of Jiangxi and Hunan Provinces and has a sub-tropical monsoon climate. It has four distinctive seasons and approximately 3200 species of vascular plants. Abundant plant resources provide a favourable environment for animals in this mountain. Currently, there are more than 2700 insect species, 270 bird species, 60 mammal species, 60 reptile species, 40 amphibian species and 40 fish species known from this area (Chen et al. 2012). However, only a few species of spiders were recorded from there before our surveys.

During the examination of the thomisid spiders collected from Jinggang Mountain National Nature Reserve in Jiangxi Province, a new species of *Ibana* was identified. This new species comprises the first of the genus described for China. Additionally, illustrations of diagnostic structures and a distribution map are presented.

## Materials and methods

Specimens were examined using a SZ6100 stereomicroscope. Epigynes were dissected, cleared in a pancreatin solution (Álvarez-Padilla and Hormiga 2007) and examined in 80% ethanol using an Olympus CX43 compound microscope with a KUY NICE CCD camera. Specimens and the dissected epigynes were preserved in 75% ethanol after examination. Types are deposited in the Animal Specimen Museum, College of Life Science, Jinggangshan University (ASM-JGSU).

The measurements were taken using an Imageview 1.0 and are given in millimetres. The body lengths of all specimens exclude the chelicerae and spinnerets. Terminology of the the female genitalia follows Benjamin (2011) and Benjamin (2014).

Leg measurements are given as: total length (femur, patella, tibia, metatarsus, tarsus). Promarginal and retromarginal teeth on the chelicerae are given as the proximal and median and counted from the base of the fang to the distal groove. The abbreviations used in the figures and text are as follows:

Eyes

- AER = anterior eye row;
- ALE = anterior lateral eye;
- AME = anterior median eye;
- MOA = median ocular area;
- PER = posterior eye row;
- PLE = posterior lateral eye;
- PME = posterior median eye.

Legs

- d = dorsal;
- Fe = femur;
- Mt = metatarsus;
- p = prolateral;
- Pt = Patella;
- r = retrolateral;
- Ta = tarsus;
- Ti = tibia;
- v = ventral.

Epigyne

- CD = copulatory duct;
- CO = copulatory opening;
- FD = fertilisation duct;
- Spe = spermatheca;
- TrC = transverse ridge of copulatory openings.

## Taxon treatments

### Ibana gan

Liu & Li sp. n.

6EB13229-ED35-593B-909F-C2153AD4EBD9

2A1D6362-7BD5-4154-B5A5-477B438C413E

#### Materials

- **Type status:** Holotype. **Occurrence:** recordedBy: Liu Ke-Ke; individualCount: 1; sex: female; lifeStage: adult; **Taxon:** scientificName: Ibanagan; **Location:** country: China; stateProvince: Jiangxi; county: Jinggangshan County Level City; locality: Jinggang Mountain National Nature Reserve, Maoping Town, Shenshan Village; verbatimElevation: 924 m; verbatimCoordinates: 26°39'22.63"N, 114°12'20.16"E; georeferenceProtocol: GPS; **Identification:** identifiedBy: Liu Ke-Ke; dateIdentified: 2022; **Event:** samplingProtocol: sweeping; eventDate: 07/04/2020
- **Type status:** Paratype. **Occurrence:** recordedBy: Liu Ke-Ke; individualCount: 2; sex: female; lifeStage: adult; **Taxon:** scientificName: Ibanagan; **Location:** country: China; stateProvince: Jiangxi; county: Jinggangshan County Level City; locality: Jinggang Mountain National Nature Reserve, Maoping Town, Shenshan Village; verbatimElevation: 924 m; verbatimCoordinates: 26°39'22.63"N, 114°12'20.16"E; georeferenceProtocol: GPS; **Identification:** identifiedBy: Liu Ke-Ke; dateIdentified: 2022; **Event:** samplingProtocol: sweeping; eventDate: 07/04/2020
- **Type status:** Paratype. **Occurrence:** recordedBy: Liu Ke-Ke; individualCount: 1; sex: female; lifeStage: adult; **Taxon:** scientificName: Ibanagan; **Location:** country: China; stateProvince: Jiangxi; county: Jinggangshan County Level City; locality: Ciping Town, Dajing Village, General Forest; verbatimElevation: 988 m; verbatimCoordinates: 26°33'3.6"N, 114°6'50.4"E; georeferenceProtocol: GPS; **Identification:** identifiedBy: Liu Ke-Ke; dateIdentified: 2022; **Event:** samplingProtocol: sweeping; eventDate: 07/03/2020

#### Description

**Figure 1.**
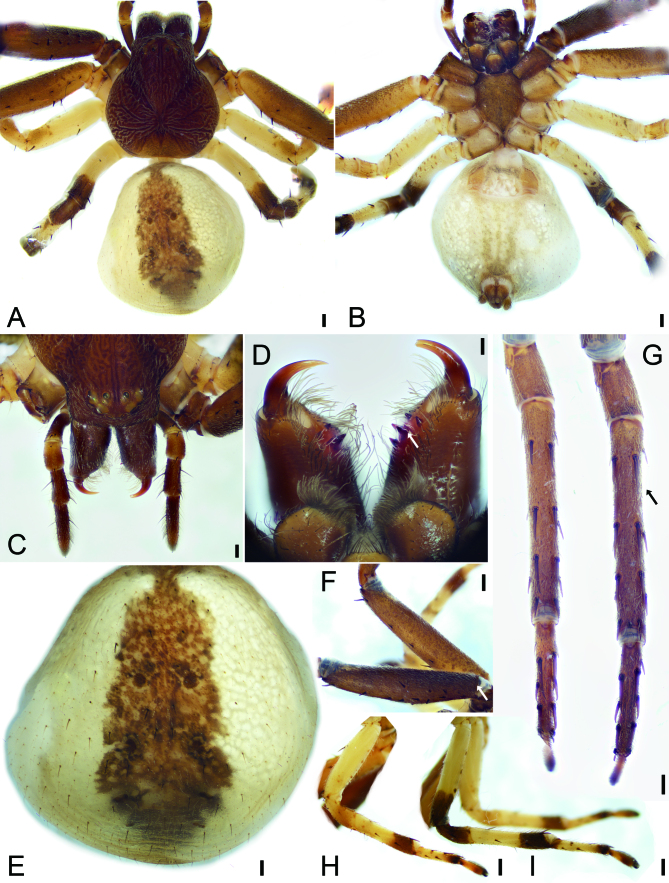
*Ibanagan* **sp. n.**, female holotype. **A** habitus, dorsal view; **B** same, ventral view; **C** frontal part of prosoma, dorsal view; **D** chelicerae, white arrow shows the distal stout retromarginal tooth, posterior view; **E** abdomen, dorsal view; **F** legs I and II, femora, white arrow shows femur I, prolateral view; **G** same, black arrow shows leg I, ventral view; **H** leg III, retrolateral view; **I** leg IV, retrolateral view. Scale bars: A, B, F–I 0.5 mm; C, D 0.1 mm, E 0.2 mm.

**Female** (holotype). Habitus as in Fig. 1A and B and Fig. 4B–D. Total length 9.5, carapace 4.1 long, 3.94 wide, with abundant white setae on surface. AER strongly recurved and PER slightly recurved; eye sizes and interdistances (Fig. 1A and C): AME 0.1, ALE 0.19, PME 0.14, PLE 0.16; AME–AME 0.17, AME–ALE 0.13, PME–PME 0.29, PME–PLE 0.25, AME–PME 0.36, AME–PLE 0.56, ALE–ALE 0.59, PLE–PLE 1.07, ALE–PLE 0.27. MOA 0.42 long, frontal width 0.34, posterior width 0.56. Chelicerae (Fig. 1B and D) with abundant setae on frontal part, three promarginal (median largest, proximal smallest) and two retromarginal teeth (distal stout, apex split into two branches of different sizes). Endites (Fig. 1B and D) slightly oblique, longer than wide, brush-shaped, anterolateral area of endite with rows of thick setae. Labium slightly wider than long, anteriorly with 12–14 setae. Sternum (Fig. 1B), longer than wide, anteromedially with a wide notch, lateral margin with weak precoxal triangles, except intercoxae I and II, posteriorly triangular, pointed end. Legs (Fig. 1F–I): measurements: I 17.41 (5.19, 2.26, 4.99, 3.26, 1.71); II 17.07 (5.24, 2.22, 4.92, 3.05, 1.64); III 9.18 (2.93, 1.47, 2.36, 1.58, 0.84); IV 10.84 (3.58, 1.53, 2.69, 2.00, 1.04); spination: I Fe: d10, p3, r5; Pa: d1, r1; Ti: p3, r3, v8; Mt: d2, p2, r2, v4; II Fe: d9; Pa: d1, r1; Ti: p3, r1, v8; Mt: d2, p2, r2, v4; III Fe: d2; Pa: d1; Ti: d3, p3, r2, v4; Mt: d3, p2, r3, v5; IV: Fe: d2, p1, r1; Pa: p1, r1; Ti: d2, p2, r2, v4; Mt: d3, p3, r1, v3. Abdomen (Fig. 1E) 5.07 long, 4.91 wide, with two pairs of sigilla in anteromedial part.

Colouration (Fig. 1). Carapace reddish-brown, with radial, irregular, narrowed dark-brown stripes around longitudinal fovea. Chelicerae dark brown. Endites and labium yellowish-brown. Sternum yellowish-brown. Legs: coxae I yellow brown, II–IV yellow; femora, patellae, tibiae, metatarsi and tarsi I and II yellow brown; leg III yellow, with dark brown annulations on distal tibia and metatarsus; leg IV yellow, with dark brown annulations on distal femur, tibia and metatarsus and on patella. Abdomen yellowish-white, medially with a broad yellow brown band; venter with three yellowish-brown stripes medially.

**Figure 2.**
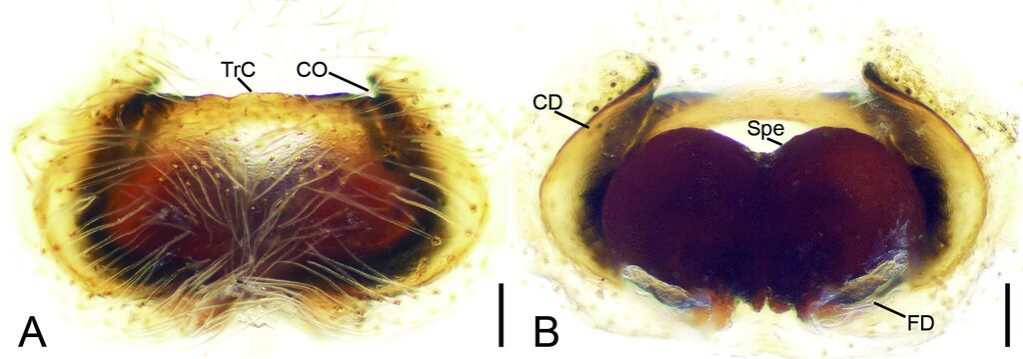
*Ibanagan* **sp. n.**, female holotype. **A** epigyne, ventral view; **B** vulva, dorsal view. Abbreviations: CD – copulatory duct, CO – copulatory opening, FD – fertilisation duct, Spe – spermatheca, TrC – transverse ridge of copulatory openings. Scale bars: 0.1 mm.

**Figure 3.**
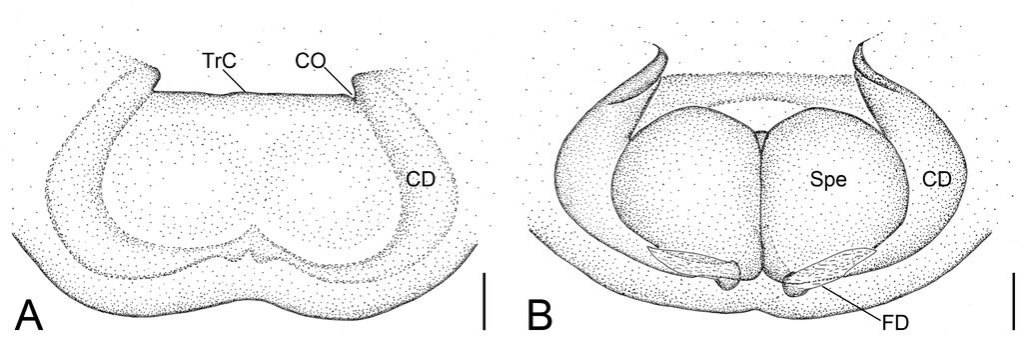
*Ibanagan* **sp. n.**, female holotype. **A** epigyne, ventral view; **B** vulva, dorsal view. Abbreviations: CD – copulatory duct, CO – copulatory opening, FD – fertilisation duct, Spe – spermatheca, TrC – transverse ridge of copulatory openings. Scale bars: 0.1 mm.

Epigyne (Fig. 2 and Fig. 3). Peento-like, anteromedially with a transverse ridge (TrC). Copulatory openings (CO) located at the lateral of the ridge, slit-like. Copulatory ducts (CD) flat, extending along spermathecae from anterolateral of the ridge to posterior of spermathecae. Spermathecae (Spe) round, contiguous. Fertilisation ducts (FD) relatively broad, located posteriorly on spermathecae, directed anterolaterally.

**Male.** Unknown.

##### Comments

**Figure 4.**
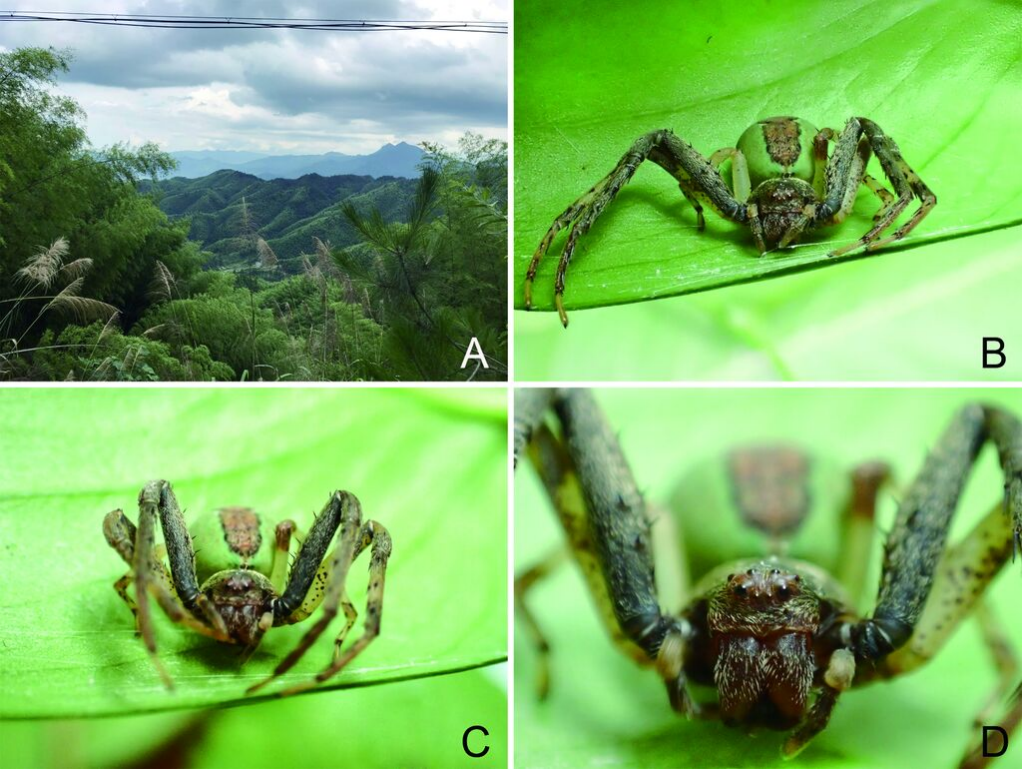
*Ibanagan* **sp. n.**, female. **A** type locality in Huangyangjie Scenic Spot, Jinggang Mountain National Nature Reserve; **B–D** photos of living specimen.

In life (Fig. 4), this species exhibits green colours in most parts of the legs and abdomen, while yellowish after preservation in ethanol.

#### Diagnosis

The new species can be distinguished from *Ibanasenagang* Benjamin, 2014 (Benjamin 2014: 181, figs. 1G, 3A–C) by the transverse ridge of copulatory openings present (vs. absent), the copulatory ducts extending from anterolateral to posteromedial part of epigyne (vs. from medial to anteromedial) and the oval, contiguous spermathecae (vs. close touching) (Figs 2, 3).

#### Etymology

The specific name refers to the Chinese abbreviation for Jiangxi Province; noun in apposition.

#### Distribution

**Figure 5.**
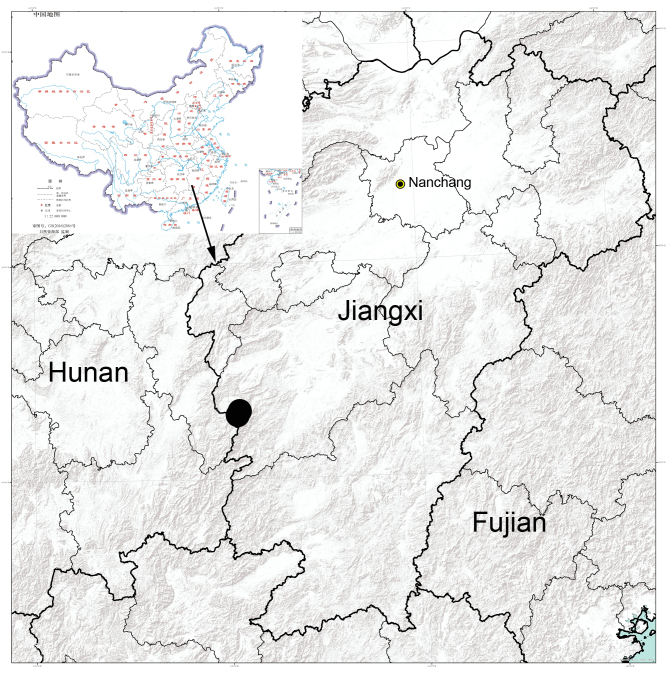
Records of *Ibanagan* **sp. n.**, from Jiangxi Province, China.

Known only from the type locality in Jiangxi Province, China (Fig. 5).

## Supplementary Material

XML Treatment for Ibana gan
